# Application of different proportions of sweet sorghum silage as a substitute for corn silage in dairy cows

**DOI:** 10.1002/fsn3.3347

**Published:** 2023-05-15

**Authors:** Xiaokang Lv, Liang Chen, Chuanshe Zhou, Guijie Zhang, Jingjing Xie, Jinhe Kang, Zhiliang Tan, Shaoxun Tang, Zhiwei Kong, Zixin Liu, Zhiyan Du

**Affiliations:** ^1^ CAS Key Laboratory for Agro‐Ecological Processes in Subtropical Region, National Engineering Laboratory for Pollution Control and Waste Utilization in Livestock and Poultry Production, Hunan Provincial Key Laboratory of Animal Nutrition Physiology and Metabolic Process Institute of Subtropical Agriculture, Chinese Academy of Sciences Changsha Hunan 410125 China; ^2^ College of Advanced Agricultural University of Chinese Academy of Sciences Beijing 100049 China; ^3^ Research Institute of Rural Revitalization Strategy Shenyang Agricultural University Shenyang Liaoning 110866 China; ^4^ School of Agriculture Ningxia University Yinchuan Ningxia 750021 China; ^5^ Hunan Longping Hi‐Tech Cultivated and Restoration Technology Co., Ltd Changsha Hunan 410125 China

**Keywords:** corn silage, dairy cows, sweet sorghum silage

## Abstract

This experiment explored the effects of different proportions of sweet sorghum silage as a substitute for corn silage on dry matter intake (DMI), milk yield, milk quality, apparent digestibility, rumen fermentation parameters, serum amino acid profile, and rumen microbial composition of dairy cows. A total of 32 mid‐lactation Holstein dairy cows with similar body weights and parities were randomly divided into four treatments: 100% corn silage +0% sorghum silage (CON), 75% corn silage +25% sorghum silage (CS1), 50% corn silage +50% sorghum silage (CS2), and 25% corn silage +75% sorghum silage (CS3). The milk yield was increased (linear, *p* = .048) as the proportion of sweet sorghum increased. Linear (*p* = .003) and quadratic (*p* = .046) increased effects were observed in milk fat as corn silage was replaced with sorghum silage. Compared with the CON diet group, the CS2 and CS3 diet groups had lower dry matter (DM) (linear, *p* < .001), ether extract (EE) (linear, *p* < .001), and gross energy (GE) (linear, *p* = .001) digestibility of the dairy cows. The ruminal fluid aspartate (Asp) level decreased (linear, *p* = .003) as the proportion of sweet sorghum increased. Linear (*p* < .05) and quadratic (*p* < .05) increased effects were observed for the contents of threonine (Thr), glycine (Gly), valine (Val), leucine (Leu), tyrosine (Tyr), and histidine (His) in rumen fluid with the replacement of corn silage with sorghum silage. Cows fed the CS3 diet had greater *Faecalibacterium, Bacteroides*, and *Prevotella ruminicola* content/copy number than those fed the CON diet (*p* < .05). In conclusion, feeding sorghum silage as a replacement for corn silage could increase the milk yield and fat, promote the growth of rumen microbes, and provide more rumen fluid amino acids for the body and microbial utilization. We believe that sorghum silage is feasible for dairy cows, and it is reasonable to replace corn silage with 75% sorghum silage.

## Graphical abstract

Feeding sorghum silage as a replacement for corn silage could increase milk yield and fat. Sorghum silage is feasible for dairy cows, and it is reasonable to replace corn silage with 75% sorghum silage.

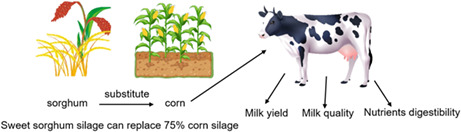

## 1 INTRODUCTION

In recent years, with the improvement of living standards and changes in dietary structure, consumer demand for ruminant livestock products has continued to increase, which has led to the continuous expansion of the scale and number of ruminant livestock breeds, resulting in a lack of roughage, especially high‐quality roughage. Annual variations in climatic conditions (e.g., rainfall, drought, etc.) are often reasons for the limited amount of forage production in numerous regions of China. Corn silage has become the main forage feed for dairy cows in many areas due to its high DM yield, high energy concentration, and optimum DM concentration at harvest for good forage fermentation and storage (Colombini et al., 2010). Nevertheless, drought, water supply, high summer temperatures, and mycotoxin contamination have brought considerable risks to corn silage production, which leads farmers to use alternative forages for ensiling (Cattani et al., 2017; Gentry et al., 2013; Vencill et al., 2012).

Forage sorghum (Fs) has been cultivated in many areas of China due to its drought and salinity tolerance, lower water requirements, and fertility requirements than corn (Contreras‐Govea et al., 2010). Among the advantages of using sorghum are the high DM yield compared with other grasses and the possibility of regrowth (Borba et al., 2012). Compared with corn silage, sorghum silage has a lower starch content but a relatively higher sugar content and a higher feed intake in dairy cows (Broderick & Radloff, 2004). Sugar is easier to digest and absorb, and increasing the soluble sugar ratio in the diet could reduce the risk of acidosis in dairy cows (Penner & Oba, 2009). Therefore, from the perspective of dairy cows' energy utilization and rumen health, sorghum silage may be better than corn silage. Compared with traditional sorghum, the stem of sweet sorghum is rich in sugar, approximately 14% depending on the variety (Freeman et al., 1973). The high sugar content of sweet sorghum and the adaptability of sorghum to a wide range of environmental conditions has prompted researchers to evaluate the potential of sweet sorghum as an alternative crop to corn silage for ruminants. Sweet sorghum and corn silage have shown comparable results in nutrient digestibility and growth performance in beef calve (Adewakun et al., 1989). A previous study has shown that sorghum silage's crude fiber and protein contents are equal to or better than those of corn silage (Dann et al., 2008). Likewise, the DM degradation rate and neutral detergent fiber (NDF) digestibility of sorghum silage are higher than corn silage (Amer et al., 2012; Corredor et al., 2009; Zhang et al., 2016). In addition, Khosravi et al. (2018) observed that cows have the same nutrient digestibility for corn and sorghum silage. No notable difference in milk yield is reported in lactating dairy cows fed either a brown midrib (BMR) sorghum forage diet or a traditional corn silage diet (Grant et al., 1995; Oliver et al., 2004). In contrast, a previous study reported that a corn silage diet (65% on dietary DM) increased milk yield and protein compared to a BMR sorghum forage diet (65% on dietary DM) (Aydin et al., 1999), but feeding a lower ratio of both forages (35% on dietary DM) for a prolonged time did not affect the milk yield and quality. In this way, scholars hold different opinions about the application of sweet sorghum on dairy cows. As such, the application effect of sweet sorghum on dairy cows still needs to be further explored. In this research, different proportions of sweet sorghum silage (Alto 601) were used to replace corn silage, and the effects of sorghum silage on cow performance, apparent digestibility of nutrients, and rumen microbial composition were explored to provide a theoretical basis for the practical application of sweet sorghum in milk production.

## 2 MATERIALS AND METHODS

### 2.1 Crops and silages

We used Alto 601 sweet sorghum variety obtained from Hunan Longping High‐tech Arable Land Restoration Technology Co., LTD, Changsha, 410,128; the variety registration number is GPD Sorghum (2018) 430148. Sweet sorghum was planted in 2018 by Hunan Youzhuo Animal Husbandry Co., LTD, Changsha, 410,128, and harvested at the milk stage of maturity. Corn (Zea mays, Jiaxi 100) was planted and harvested simultaneously during the milk stage of maturity. Sweet sorghum and corn are directly chopped and cut to a theoretical chopping length of 2–3 cm, then placed in the ground silage, compacted with a compactor machine, and covered with plastic sheets on top. Three grams of *Lactobacillus* dry powder (5 × 10^5^ cfu/g), 3 kg of NaCl, and 2.5 kg of urea were evenly sprayed on each ton of silage after being dissolved in water. Silage samples were taken at four different points in the silage cellar, dried at 65°C, crushed, passed through a 40‐mesh sieve, and then stored in sealed plastic containers at 4°C until further analysis (Horwitz & Latimer, 2005). The nutritional compositions of sorghum and corn silage are presented in Table 1.

**TABLE 1:** Nutrient composition of sorghum silage and corn silage (% of DM).

Items	Sorghum silage	Corn silage
DM	21.24	24.65
CP	8.68	6.64
EE	2.03	3.51
PDF	64.52	53.58
ADF	37.20	24.39
NFC^a^	19.64	32.85
Starch	2.31	24.63
Ash	5.13	3.42
Lignin	4.63	3.11

Abbreviations: ADF, acid detergent fiber; CP, crude protein; DM, dry matter; EE, ether extract; NDF, neutral detergent fiber; NFC, nonfiber carbohydrate.

^a^ NFC = 100−NDF−CP−EE−Ash.

### 2.2 Animals and diets

This research was conducted at Hunan Youzhuo Animal Husbandry Co., LTD, Changsha, 410,128 (North latitude N28°24′20.90″ east longitude E112°39′46.12″). The experimental period is from June to August 2019. All animals were cared for according to procedures approved by the Institute of Subtropical Agriculture, Chinese Academy of Sciences Ethics Committee. Thirty‐two mid‐lactation Holstein dairy cows with similar body weights (595 ± 40 kg) and parities (2 ± 1.2) were randomly divided into four treatments: 100% corn silage +0% sorghum silage (CON), 75% corn silage +25% sorghum silage (CS1), 50% corn silage +50% sorghum silage (CS2), and 25% corn silage +75% sorghum silage (CS3). The composition and nutritional levels of the four different diets are shown in Table 2. Cows were fed at 6:30, 13:30, and 19:30 daily to yield approximately 5–10% refusals; water and feed were provided ad libitum during the whole experiment. The diets were formulated to meet or exceed the NRC (2001) requirements for NEL and MP of a cow with 650 kg BW, 44 kg/d MY, 3.8% fat, 3.2% true protein, and 27 kg/d DMI (Table 2). The adjustment period lasted 10 days, and the experimental period lasted 60 days. The cows were milked three times daily at 07:00, 14:00, and 20:00 h.

**TABLE 2:** Diet composition and nutritional levels of the four treatments (% of DM).

Item	Diet^a^			
	CON	CS1	CS2	CS3
Ingredient, % of DM				
Corn	14	14	14	14
Soybean meal	5.13	4.51	5.4	5.04
Sugar beet pulp	4.29	4.24	4.33	5.32
Cottonseed	4.13	4.56	4.13	4.06
Beer lees	9.47	9.26	9.56	8.69
Oat grass	4.13	4.28	4.1	3.97
Alfalfa	6.44	6.84	6.5	6.39
Corn silage	48.19	36.14	24.1	12.05
Sorghum silage	0	12.05	24.09	36.14
Molasses	1.31	1.25	1.12	0.92
Salt	0.5	0.5	0.5	0.5
Premix^b^	1	1	1	1
Fat supplement	1.41	1.37	1.17	1.92
Total	100	100	100	100
Nutrient, % of DM				
NDF	47.03	52.11	53.70	51.56
ADF	17.37	22.44	23.24	23.79
Ash	6.19	7.28	6.52	6.49
CP	13.08	13.97	13.81	13.80
EE	4.67	3.41	3.55	3.89
NFC^b^	29.03	23.23	22.42	24.26

Abbreviations: ADF, acid detergent fiber; CP, crude protein; DM, dry matter; EE, ether extract; NDF, neutral detergent fiber; NFC, nonfiber carbohydrate.

^a^ CON: 0% sorghum silage +100% corn silage; CS1: 25% sorghum silage +75% corn silage; CS2: 50% sorghum silage +50% corn silage; CS3: 75% sorghum silage +25% corn silage.

^b^ NFC = 100−NDF−CP−EE–Ash.

### 2.3 Feed intake, milk production, and milk composition

The feed and residue of total mixed ration (TMR) were recorded daily and stored at −20°C. The DM content of TMR and residues were analyzed weekly, and the intake of each cow was calculated. Cows were milked three times daily, and the amount of milk produced for each cow at each milking was recorded. Daily milk production was recorded using the Afimilk system (Kibbutz Afikim, Israel). Milk samples were collected on the 7th, 14th, 21st, and 28th days of the experimental period (50‐mL milk was taken at 07:00, 14:00, and 20:00 h and mixed at 4:3:3) to analyze milk composition. The collected milk sample was divided into two parts: one was supplemented with 2‐bromo‐2‐nitro‐1,3‐propanediol and used for milk fat, protein, and lactose determination; the other was frozen at −20°C, and the urea nitrogen in milk was determined. The milk fat, protein, lactose, total solids (TS), and nonfat solids (SNF) concentrations were determined on an infrared milk analyzer (Milko Scan FT 20076150, Foss Electric, Hillerod, Denmark). The yield of 4% FCM (kg/d) was calculated (National Research Council, 2001) as 0.4 × milk yield (kg/d) + 15.0 × fat yield (kg/d).

### 2.4 Chemical analyses of feed samples

Feeds were sampled weekly throughout the experimental period, and the DM content was determined by drying at 65°C to a constant weight. TMRs were analyzed for ash, crude protein (CP), ether extract (EE), neutral detergent fiber (NDF), and acid detergent fiber (ADF) according to the Association of Official Analytical Chemists (Horwitz & Latimer, 2005). The gross energy of the diets was determined by an isothermal automatic calorimeter (5E‐AC8018, Changsha Kaiyuan Instruments Co., Ltd, China).

### 2.5 Collection of fecal samples and determination of digestibility

Fecal samples were collected 1 week before the end of the experiment. For digestibility determination, fecal grab samples were collected from the rectum of each cow. Fecal samples (approximately 100 g) were collected eight times over 3 days at (day 1) 05:00, 12:00, and 18:00 h; (day 2) 00:00, 09:00, 15:00, and 21:00 h; and (day 3) 0300 h to obtain a representative sample of a 24‐h period. Fecal samples from each cow were composited by combining approximately 100 g of feces from each time point. Fecal samples were divided into two portions, with one portion being treated with 10% H_2_S0_4_ to determine the CP content and the other portion left untreated to determine EE, NDF, and ADF content. Samples were frozen at −20°C and dried in a forced‐air oven at 65°C for 72 h. Fecal samples were analyzed for ash, CP, EE, NDF, and ADF according to the Association of Official Analytical Chemists (Horwitz & Latimer, 2005). Feeds and fecal samples were analyzed for acid‐insoluble ash to estimate digestion (Vankeulen & Young, 1977).

### 2.6 Serum amino acid profile

On the 59th day of the experimental period, before morning feeding and 3 hours after feeding, we collected blood from the coccygeal vein of each cow. Approximately 10 mL of blood was centrifuged at 3000 *g* at 4°C for 15 min to obtain the serum. The collected serum was placed at −20°C until further analysis. The free amino acid profiles of plasma samples were determined according to the method described by Zhu et al. (2018).

### 2.7 Rumen fermentation parameters and AA profile

Rumen fluid samples were collected on day 59 of the experimental period at 3 h and 6 h after morning feeding. Rumen fluid was collected via an oral stomach tube with an attached strainer (1 mm pores). Approximately 200 mL of the initial collection of rumen fluid was discarded. After the initial volume was discarded, an additional 500 mL of rumen fluid was collected for further analysis. The volatile fatty acids (VFA) and NH_3_–N contents were determined according to the method described by Chen et al. (2017). Microbial proteins were analyzed according to the method described by Makkar et al. (1982). One milliliter of rumen fluid was added to 1 mL of 8% sulfosalicylic acid solution to precipitate protein in rumen fluid and then analyzed by high‐performance liquid chromatography using an automatic AA analyzer L‐8800 (Hitachi, Ltd, Tokyo, Japan) equipped with a column (diameter 4.6 mm and particle size 60 mm, filler: 2622).

### 2.8 DNA extraction and quantification of functional bacterial groups

According to the manufacturer's instructions, total DNA was extracted from the rumen fluid samples using the QIAamp®Fast DNA Stool Mini Kit (Qiagen, Germany). The quality and quantity of DNA were measured using a NanoDrop ND1000 (NanoDrop Technologies, Inc., Wilmington, DE, USA). The copy numbers of bacterial DNA genes were quantified using an ABI 7900 Sequence Detection System (Applied Biosystems, Foster City, CA, USA) as detailed in a previous study (Jiao et al., 2016). The values were converted to log 10 for further statistical analysis.

### 2.9 Statistical analysis

For nutrient digestion, data were analyzed using the PROC MIXED of SAS (version 9.1, SAS Institute Inc., Cary, NC). The model used for data analysis is as follows:

(1) $$Y_{\mathrm{ij}}=\mu +T_{i}+e_{\mathrm{ij}}$$

where *Y*_*ij*_ is the dependent variable, μ is the overall mean, *T*_*i*_ (*i* = 1–4) is the fixed effect of treatment, *e*_*ij*_ is the random residual error, and animals were considered random effects.

For milk production and composition, rumen fermentation parameters, and AA profiles, the following model was used:

(2) $$Y_{\mathrm{ijk}}=\mu +T_{i}+S_{j}+{\mathrm{TS}}_{\mathrm{ij}}+e_{\mathrm{ijk}}$$

where *Y*_*ijk*_ is the dependent variable, μ is the overall mean, *T*_*i*_ (*i* = 1–4) is the fixed effect of treatment, *S*_*j*_ is the sample time, *TS*_*ij*_ is the interaction effect between *T*_*i*_ treatment and *S*_*j*_ sample time, and *e*_*ijk*_ is the random residual error. Sample time was considered a repeated measure, and animals were considered a random effect. Orthogonal polynomial contrasts were used to analyze the linear and quadratic effects of sorghum silage levels. The IML procedure of SAS (SAS Inst. Inc., Cary, NC) was conducted to correct the contrast coefficients of the orthogonal polynomial.

Differences in rumen microbial population among four treatments were analyzed using the Kruskal–Wallis test, followed by multiple comparisons. A *p* value <.05 was considered to indicate a statistically significant difference.

## 3 RESULTS

### 3.1 Dry matter intake, milk yield, and composition

The effect of various substitution ratios of sweet sorghum silage on the milk quality of dairy cows is shown in Table 3. As the substituted proportion of sweet sorghum increased, the DMI increased (linearly, *p* < .001 and quadratically, *p* = .043). As the proportion of sweet sorghum increased, the milk yield increased (linear, *p* = .048). Linear (*p* = .003) and quadratic (*p* = .046) increased effects were observed in milk fat as corn silage replaced sorghum silage. Linear (*p* = .024) and quadratic effects (*p* < .001) were observed, with an increase in the 4% FCM yield when the sweet sorghum replaced the corn.

**TABLE 3:** Effect of substituting sorghum silage with different ratios for corn silage on milk quality of cows.

Item	Diet^1^				SEM	*p‐*Value		
	CON	CS1	CS2	CS3		*T*	*L*	*Q*
DMI, kg/d	18.46^b^	18.48^b^	20.13^a^	19.46^a^	0.745	<.001	<.001	.043
Milk yield, kg/d	20.81^b^	20.36^b^	22.12^a^	21.38^ab^	0.183	.032	.048	.069
Milk/DMI	1.13	1.10	1.10	1.10	0.023	.441	.132	.211
Milk fat, %	3.63^b^	3.93^a^	3.94^a^	3.63^b^	0.131	.008	.003	.046
Milk protein, %	3.43	3.42	3.64	3.48	.069	.452	.493	.838
Milk lactose, %	4.63	4.45	4.71	4.62	.074	.679	.714	.776
MUN, mg/dL	19.11	17.37	20.87	20.08	.503	.832	.443	.639
4% FCM, kg/d	19.66^b^	20.15^ab^	21.92^a^	20.19^ab^	.413	.043	.024	<.001

Abbreviations: 4% FCM, 4% fat‐corrected milk; DMI, dry matter intake; *L*, linear; MUN, milk urea nitrogen; *Q*, quadratic; *T*, treatment.

^1^ CON: 0% sorghum silage +100% corn silage; CS1: 25% sorghum silage +75% corn silage; CS2: 50% sorghum silage +50% corn silage; CS3: 75% sorghum silage +25% corn silage.

^a, b^ Means within a row with different superscripts differ (*p* < .05).

### 3.2 Nutrient apparent digestibility

The effects of replacing corn silage with different proportions of sorghum silage on the apparent digestibility of nutrients in cows are shown in Table 4. The inclusion of sweet sorghum in the dairy cow diet declined the apparent digestibility of DM (linear, *p* < .001). Compared with the CON group, the DM digestibility in the CS3 group was reduced by 4.50% (*p* < .05). A quadratic effect (*p* = .001) was observed on the apparent digestibility of ADF as sorghum silage was replaced for corn silage. Cows fed the CS2 and CS3 diets had lower GE (linear, *p* = .001) and EE digestibility (linear, *p <* .001) than those fed the CON and CS1 diets.

**TABLE 4:** Nutrients apparent digestibility of cows fed diets containing different replacement levels of corn silage with sorghum silage.

Item	Diet^1^				SEM	*p*‐Value		
	CON	CS1	CS2	CS3		*T*	*L*	*Q*
DM	77.94^a^	76.91^a^	73.95^b^	74.43^b^	0.490	<.001	<.001	.277
NDF	75.60	74.20	73.66	73.98	0.531	.363	.287	.447
ADF	59.52^b^	67.46^a^	61.14^ab^	62.53^ab^	0.975	.021	.164	.001
GE	78.99^a^	78.34^a^	75.18^b^	75.68^b^	0.498	.003	.001	.432
EE	82.45^a^	81.49^a^	72.11^b^	75.16^b^	1.138	<.001	<.001	.426
CP	74.77	76.99	73.03	77.35	0.537	.320	.297	.196

Abbreviations: ADF, acid detergent fiber; CP, crude protein; DM, dry matter; EE, ether extract; GE, gross Energy; L, linear; NDF, neutral detergent fiber; Q, quadratic; T, treatment.

^1^ CON: 0% sorghum silage +100% corn silage; CS1: 25% sorghum silage +75% corn silage; CS2: 50% sorghum silage +50% corn silage; CS3: 75% sorghum silage +25% corn silage.

^a, b^ Means within a row with different superscripts differ (*p* < .05).

### 3.3 Rumen fermentation

Table 5 provides the effects of replacing corn silage with different ratios of sweet sorghum silage on rumen fermentation parameters in dairy cows. When the levels of sweet sorghum were increased in the diets, the proportion of acetate (linear, *p* < .001) decreased. A decreased effect (quadratic, *p* = .014) was observed for the proportion of propionate with the replacement of corn silage by sorghum silage. Cows fed a diet with high levels of sweet sorghum had a higher proportion of valerate (linear, *p* = .001). The acetate: propionate (A:P) was decreased quadratically (*p* = .007) as the substitution ratio of the sweet sorghum increased. The content of ruminal TVFA was increased (linear, *p* = .042) with increasing sweet sorghum in the diet. Increased effects (linear and quadratic, *p* < .001) were observed for the contents of butyrate, isobutyrate, and isovalerate with the replacement of corn by sweet sorghum. As the proportion of sweet sorghum increased, the content of NH_3_–N decreased (linear, *p* = .034).

**TABLE 5:** Rumen fermentation parameters of cows fed diets containing different replacement levels of corn silage with sorghum silage.

Item	Diet^1^				SEM	*p‐*Value		
	CON	CS1	CS2	CS3		*T*	*L*	*Q*
TVFA, mmol/L	54.96^b^	73.24^a^	78.21^a^	75.73^a^	3.860	.024	.042	.150
Acetate, %	72.34^a^	65.00^b^	61.39^b^	60.27^b^	0.011	<.001	<.001	.097
Propionate, %	24.29^a^	19.24^b^	20.92^ab^	22.09^ab^	0.007	.016	.264	.014
Valerate, %	0.02^b^	1.40^a^	1.60^a^	1.59^a^	0.001	.003	.001	.409
Isovalerate, %	1.69^b^	2.24^a^	1.99^ab^	2.10^a^	0.001	<.001	<.001	<.001
Butyrate, %	0.81^c^	11.15^b^	12.87^a^	12.94^a^	0.011	<.001	<.001	<.001
Isobutyrate, %	0.85^b^	0.97^b^	1.23^a^	1.01^b^	0.001	<.001	<.001	<.001
Acetate/Propionate	2.98^ab^	3.38^a^	2.93^ab^	2.73^b^	0.112	.001	.067	.007
NH_3_–N (mg/dL)	13.65^a^	12.24^a^	10.67^ab^	8.84^b^	0.270	.021	.034	.848
MCP(mg/mL)	8.36	8.13	8.41	9.89	0.294	.060	.054	.135

Abbreviations: *L*, linear; MCP, microbial protein; *Q*, quadratic; *T*, treatment; TVFA, total volatile acid.

^1^ CON: 0% sorghum silage +100% corn silage; CS1: 25% sorghum silage +75% corn silage; CS2: 50% sorghum silage +50% corn silage; CS3: 75% sorghum silage +25% corn silage.

^a, b^ Means within a row with different superscripts differ (*p* < .05).

### 3.4 Amino acid profile of rumen fluid

Table 6 presents the effect of substituting corn silage with various ratios of sweet sorghum silage on the AA profile of the rumen fluid in dairy cows. As the proportion of sweet sorghum increased, the level of Asp decreased (linear, *p* = .003), and the treatment CS3 was significantly lower than other treatments. In contrast, the levels of serine (Ser), isoleucine (Ile), phenylalanine (Phe), proline (Pro), and total amino acids (TAA) increased linearly (*p* < .05) as the sweet sorghum proportion increased. Adding sweet sorghum to the diet increased (linear and quadratic, *p* < .05) the contents of Thr, Gly, Val, Leu, Tyr, and His in rumen fluid. Neither alanine (Ala) and methionine (Met) nor lysine (Lys) (*p* > .05) were affected by the treatments.

**TABLE 6:** Effect of substituting sorghum silage with different ratios for corn silage on the amino acid profile of rumen fluid of cows.

Item^1^	Diet^2^				SEM	*p*‐Value		
	CON	CS1	CS2	CS3		*T*	*L*	*Q*
Asp	5.43^a^	4.41^a^	5.01^a^	2.60^b^	0.315	.020	.003	.189
Thr	3.70^b^	3.93^b^	4.80^b^	7.50^a^	0.422	.006	<.001	.032
Ser	3.06^c^	3.75^bc^	4.58^b^	6.92^a^	0.357	<.001	<.001	.083
Glu	20.65^b^	22.53^b^	33.47^a^	12.60^c^	1.980	<.001	.277	<.001
Gly	5.25^b^	6.24^b^	6.87^b^	18.59^a^	1.175	<.001	<.001	<.001
Ala	22.65	28.50	25.08	25.74	2.828	.455	.829	.669
Val	7.73^b^	9.14^b^	8.01^b^	22.89^a^	1.670	<.001	<.001	.010
Met	1.55	1.76	1.75	1.59	0.112	.665	.927	.438
Ile	3.32^b^	5.06^b^	5.17^b^	9.92^a^	0.665	<.001	<.001	.199
Leu	6.66^b^	8.01^b^	8.15^b^	19.96^a^	1.472	.015	.001	.027
Tyr	2.37^c^	3.16^bc^	3.53^b^	8.24^a^	0.441	<.001	<.001	<.001
Phe	2.49^b^	3.42^b^	3.20^b^	6.64^a^	0.437	.033	.042	.091
Lys	12.20	12.20	13.86	11.16	0.699	.739	.820	.361
His	1.05^b^	1.10^b^	1.17^b^	6.17^a^	0.491	<.001	<.001	<.001
Arg	–	–	–	17.15	–	–	–	–
Pro	2.56^c^	3.94^c^	6.69^b^	9.95^a^	0.720	<.001	<.001	.526
TAA	100.33^b^	110.81^b^	130.17^b^	176.39^a^	9.415	.008	.002	.276

*Note*: The content of Arg in the rumen fluid of the CON, CS1, and CS2 groups was not detected.

Abbreviations: *L*, linear; *Q*, quadratic; *T*, treatment; TAA, total amino acids.

^1^ (μg/mL).

^2^ CON: 0% sorghum silage +100% corn silage; CS1: 25% sorghum silage +75% corn silage; CS2: 50% sorghum silage +50% corn silage; CS3: 75% sorghum silage +25% corn silage.

^a, b, c^ Means within a row with different superscripts differ (*p* < .05).

### 3.5 Serum biochemical indicators and amino acid profiles

Table 7 shows the effect of substituting corn silage with different ratios of sweet sorghum silage on the serum biochemical indices of dairy cows. A quadratic decreased effect was observed for the serum TG (*p* = .001) content as corn silage was replaced with sorghum silage. The level of NH_3_L increased (quadratic, *p* < .001) as the proportion of sweet sorghum increased. Linear (*p* < .001) and quadratic (*p* = .032) increased effects were observed for the content of serum Asp with the replacement of corn by sweet sorghum silage (Table 8). With the increase in the addition of sweet sorghum to the diet, the contents of serum Glu, Ala, Val, Ile, Leu, Tyr, His, Pro, and TAA showed a linear decrease (*p* < .05) (Table 8). The contents of serum Thr, Gly, Met, Ser, Phe, Lys, and Arg were similar among all treatments (*p* > .05) (Table 8).

**TABLE 7:** Effect of substituting sorghum silage with different ratios for corn silage on serum biochemical indexes of cows.

Item	Diet^1^				SEM	*p‐*Value		
	CON	CS1	CS2	CS3		*T*	*L*	*Q*
TP g/L	72.91	74.46	78.19	77.44	0.98	.055	.050	.652
GLU mmol/L	3.47	3.49	3.34	3.48	0.04	.882	.690	.420
TG mmol/L	0.21^a^	0.18^b^	0.17^b^	0.19^ab^	0.01	.043	.086	.001
CHOL mmol/L	3.82	3.82	3.47	4.11	0.15	.654	.711	.293
NH_3_L μmol/L	66.37^b^	90.27^a^	89.21^a^	74.44^b^	2.75	.030	.297	<.001

Abbreviations: CHOL, cholesterol; GLU, glucose; *L*, linear; *Q*, quadratic; *T*, treatment; TG, triglyceride; TP, total protein.

^1^ CON: 0% sorghum silage +100% corn silage; CS1: 25% sorghum silage +75% corn silage; CS2: 50% sorghum silage +50% corn silage; CS3: 75% sorghum silage +25% corn silage.

^a, b, c^ Means within a row with different superscripts differ (*p* < .05).

**TABLE 8:** Effect of substituting sorghum silage with different ratios for corn silage on serum amino acid profile of cows.

Item^1^	Diet^2^				SEM	*p*‐Value		
	CON	CS1	CS2	CS3		*T*	*L*	*Q*
Asp	0.59^d^	0.85^c^	1.12^b^	1.57^a^	0.081	<.001	<.001	.032
Thr	10.80	9.57	9.50	9.70	0.373	.561	.634	.796
Ser	10.12	8.21	8.64	8.59	0.279	.066	.074	.081
Glu	10.45^a^	9.97^ab^	8.41^bc^	8.46^c^	0.254	.004	.001	.255
Gly	24.11	21.94	21.16	23.18	0.744	.288	.595	.189
Ala	23.36^a^	22.84^ab^	20.70^ab^	19.20^b^	0.673	.020	.014	.694
Val	40.17^a^	35.45^ab^	32.14^b^	30.50^b^	1.192	.005	.001	.429
Met	1.99	1.67	1.71	1.79	0.055	.088	.238	.071
Ile	17.20^a^	15.68^ab^	15.53^ab^	14.25^b^	0.485	.031	.047	.897
Leu	34.94^a^	31.53^a^	28.56^b^	25.22^b^	1.006	<.001	<.001	.978
Tyr	14.72^a^	12.07^b^	10.52^b^	9.53^b^	0.598	<.001	<.001	.369
Phe	10.86	9.45	8.84	9.81	0.308	.110	.148	.057
Lys	10.36	9.38	9.53	9.74	0.261	.313	.499	.651
His	11.05^a^	10.17^ab^	9.59^ab^	8.56^b^	0.330	.038	.049	.651
Arg	26.53	24.65	25.64	27.53	0.560	.269	.452	.109
Pro	15.44^a^	13.13^ab^	12.97^ab^	9.94^b^	0.640	.009	.003	.740
TAA	253.84^a^	226.70^ab^	209.59^b^	198.45^b^	6.671	.026	.037	.256

Abbreviations: *L*, linear; *Q*, quadratic; *T*, treatment; TAA, total amino acids.

^1^ (μg/mL).

^2^ CON: 0% sorghum silage +100% corn silage; CS1: 25% sorghum silage +75% corn silage; CS2: 50% sorghum silage +50% corn silage; CS3: 75% sorghum silage +25% corn silage.

^a, b, c, d^ Means within a row with different superscripts differ (*p* < .05).

### 3.6 Rumen functional bacterial groups

Table 9 provides the summary statistics for the differences in the rumen microbial population among the four treatments. It was apparent from this table that cows fed the CS3 diet had a greater content of *Faecalibacterium, Prevotella*, *and Prevotella ruminicola* than those fed the CON diet (*p* < .05). The methanogen content in the CS1 group was significantly reduced compared with that in the CON and CS3 groups (*p* < .05). However, the contents of the general bacteria, *Ruminococcus albus*, *Fibrobacter succinogenes*, *Selenomonas ruminantium*, *Streptococcus bovis*, and *Ruminobacter amylophilus* were not altered by the different diets (*p* > .05).

**TABLE 9:** Copy numbers (log10(copies/g digesta)) of 16S rRNA genes of bacterial genera in the rumen digesta.

Items	All samples (*n* = 28)	Diet^a^				Kruskal–Wallis Test, *p‐*value	Multiple Comparison^a^
		CON (*n* = 8)	CS1 (*n* = 8)	CS2 (*n* = 6)	CS3 (*n* = 6)		
General bacteria	10.64 ± 0.31	10.75 ± 0.12	10.62 ± 0.13	10.55 ± 0.41	10.59 ± 0.51	.257	–
Methanogen	8.84 ± 0.26	9.03 ± 0.24	8.63 ± 0.14	8.72 ± 0.25	9.00 ± 0.13	.003	CON–CS1
							CS3–CS1
Faecalibacterium	6.03 ± 0.23	5.88 ± 0.09	5.99 ± 0.20	5.95 ± 0.11	6.37 ± 0.11	.001	CS3–CON
							CS3–CS2
Bacteroides	10.41 ± 0.16	10.34 ± 0.11	10.37 ± 0.17	10.37 ± 0.10	10.58 ± 0.13	.042	CS3–CS1
							CS3–CS1
Ruminococcus albus	7.06 ± 0.22	7.18 ± 0.18	6.98 ± 0.25	6.92 ± 0.21	7.16 ± 0.12	.127	–
Butyrivibrio fibrisolvens	9.95 ± 0.10	10.51 ± 0.11	8.52 ± 0.73	10.43 ± 0.08	10.61 ± 0.09	<.0001	CON–CS1
							CS3–CS1
Fibrobacter succinogenes	8.12 ± 0.16	8.23 ± 0.18	8.10 ± 0.11	7.99 ± 0.19	8.15 ± 0.06	.074	–
Selenomonas ruminantium	9.48 ± 0.24	9.54 ± 0.23	9.34 ± 0.23	9.45 ± 0.25	9.61 ± 0.18	.233	–
Clostridial	10.02 ± 0.19	10.10 ± 0.15	9.87 ± 0.19	9.98 ± 0.16	10.14 ± 0.12	.023	CS3–CS1
Streptococcus bovis	7.33 ± 0.21	7.46 ± 0.19	7.21 ± 0.20	7.34 ± 0.14	7.32 ± 0.26	.106	–
Prevotella	10.86 ± 0.23	10.75 ± 0.23	10.84 ± 0.23	10.78 ± 0.11	11.10 ± 0.16	.026	CS3–CS2
							CS3–CON
							CS3–CS1
Ruminococcus flavefaciens	7.66 ± 0.22	7.76 ± 0.16	7.54 ± 0.20	7.48 ± 0.17	7.87 ± 0.10	.003	CS3–CS2
							CS3–CS1
Ruminobacter amylophilus	7.00 ± 0.35	6.95 ± 0.46	7.06 ± 0.43	6.95 ± 0.29	7.03 ± 0.20	.811	–
Prevotella ruminicola	9.35 ± 0.18	9.22 ± 0.14	9.34 ± 0.16	9.39 ± 0.15	9.50 ± 0.20	.031	CS3–CON

^a^ CON: 0% sorghum silage +100% corn silage; CS1: 25% sorghum silage +75% corn silage; CS2: 50% sorghum silage +50% corn silage; CS3: 75% sorghum silage +25% corn silage.

**FIGURE 1:**
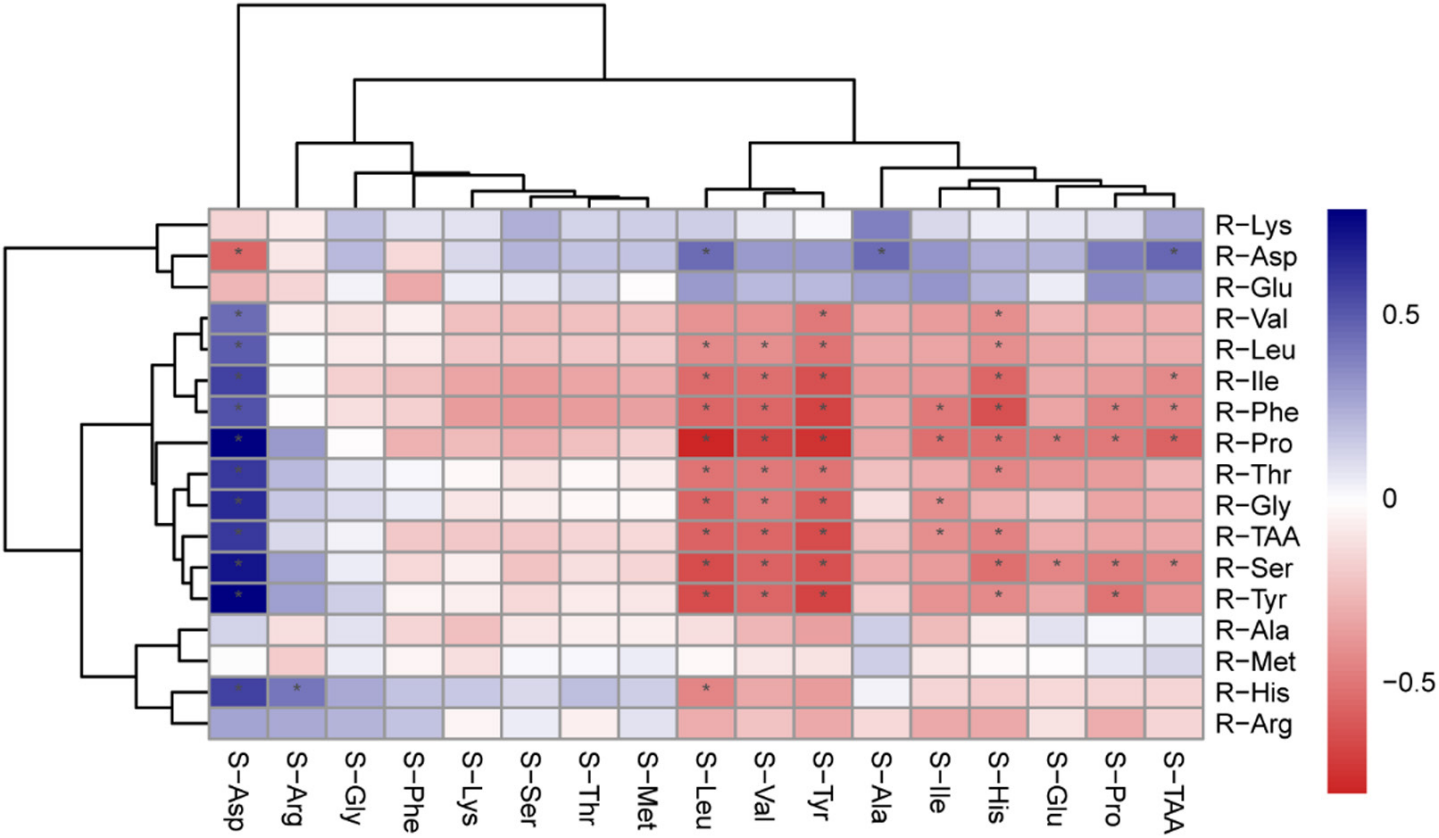
Heat map summarizing the correlation of serum AA profile and rumen fluid AA profile. Red cells represent the positive correlation coefficient and blue cells represent the negative coefficient. R, rumen fluid; S, serum.

Figure 1 shows the correlation analysis of the amino acid profile of serum and rumen fluid. The serum Leu, Val, and Tyr showed a negative correlation (*p* < .05) with the rumen fluid Leu, Ile, Phe, Pro, Thr, Gly, TAA, Ser, and Tyr. However, there was a significant positive correlation (*p* < .05) between the serum Asp and the rumen fluid Val, Leu, Ile, Phe, Pro, Thr, Gly, TAA, Ser, Tyr, and His.

## 4 DISCUSSION

### 4.1 Dry matter intake, milk yield, and composition

Due to the high yield of sorghum and its strong ability to adapt to drier environments, many scholars are devoted to exploring sorghum as a substitute for corn silage in dairy production (Peerzada et al., 2017). We found that the DMI of cows increased as the proportion of sorghum silage in the cow's diet was raised. Shaver et al. (Shaver, 2010) noted that the cows fed a diet containing the sorghum silage diet showed higher DMI than those fed a diet containing corn silage, which is similar to our findings. Conversely, a previous study showed that cows in the sorghum silage group showed lower DMI than that in the corn silage group (Harper et al., 2017). The increase in DMI of cows fed sorghum silage may be attributed to the higher ADF digestibility of sorghum silage, which can reduce the gastrointestinal filling of cows (Oba & Allen, 1999), thereby increasing space for more intake. The present data also indicated that cows consuming sweet sorghum are able to increase milk production and fat. In this study, the increased milk production of the cows fed sweet sorghum silage may be due to greater feed intake in this group. DMI is an essential factor affecting milk production, and the increase in DMI can meet the higher milk production demand of dairy cows (Khosravi et al., 2018). We also found that cows fed sorghum silage diets had a higher milk fat than those fed corn silage. Previous research declared that a higher propionate concentration in the rumen could inhibit the transport of milk fat precursors to the mammary gland, resulting in a decrease in milk fat synthesis (Doreau et al., 1999). In this study, the propionate concentration in the rumen fluid was higher in cows from the CON group (Table 3), which may be the reason for the higher milk fat content of cows fed a sweet sorghum silage diet compared with a corn silage diet. We also found that when 75% of corn silage in the diet was replaced by sorghum silage, 4% FCM was the highest, and the CON group had the lowest 4% FCM yield. This may be due to the higher DMI of cows in the CS1, CS2, and CS3 groups compared with the CON group. Increasing the energy intake of cows could satisfy the energy demand of cows during lactation, thereby elevating the 4% FCM yield.

### 4.2 Nutrient apparent digestibility

The current study suggested that the ratio of sorghum silage to corn silage in the cow diet exceeded 50%, and the apparent digestibility of DM, GE, and EE was reduced, which might be related to the high NDF content in the sorghum silage diet. The high content of NDF in the diet negatively correlates with the digestibility of nutrients. As animals consume diets with high NDF, they must increase their feed intake to meet their nutritional requirements, which means that more feed passes through the gastrointestinal tract, ultimately reducing digestibility. A further substantial cause may be that the lignin content of sorghum silage is 48.87% higher than that of corn silage, and lignin is difficult to digest by animals (Soest, 1994), resulting in a decrease in the apparent digestibility of GE, DM, and EE after cows consume sorghum silage. In line with this observation, providing a sorghum diet reduced the nutrient digestibility of dairy cows, as previously reported by Harper et al. (2017). In a study by Yang et al. (2019), no difference was observed in the digestibility of a corn and sorghum silage diet. The discrepancy between studies may be sourced from different sorghum varieties used in the studies. There was no difference in the apparent digestibility of CP among treatments. No differences in CP digestibility have been reported for cows fed sorghum silage or corn silage (Oliver et al., 2004).

### 4.3 Rumen fermentation

In this case, our results show that the ratio of acetic to propionic acid was decreased in the rumen of dairy cows from the sorghum silage group compared with the corn silage group. Corn silage has a lower NDF and higher NFC content than sorghum silage. Ordinarily, corn can quickly ferment and produces more propionic acid in the rumen due to its higher NFC content than sorghum silage. Similar to our findings, studies showed that sorghum silage reduced propionic acid content in the rumen compared to corn silage (Dann et al., 2008; Sarubbi et al., 2014; Yang et al., 2001). A previous study declared that when the digestible carbohydrates in the diet increased, the proportion of propionic acid in the rumen also increased (Yang et al., 2001). The A:P of all treatments is higher than 2.0, and an A:P <2.0 is related to milk fat depression (Al‐Suwaiegh et al., 2002). This shows that neither corn silage nor sorghum silage will reduce milk fat synthesis in cows. We found the highest A:P value of rumen fluid in the CS1 group. The highest A:P ratio for cows fed the CS1 diet corresponds well with the greatest milk fat and 4% FCM yield. In our study, the NH_3_–N content of all treatment groups was above 5 mg/dL, revealing that sorghum and corn silage could support the NH_3_–N required for optimal microbial growth (Satter & Slyter, 1974). Feeding a diet containing sorghum silage decreased the ruminal NH3–N content in this study, consistent with the previous study that showed that NH3–N concentration was lower in the rumen of steers fed a diet containing sorghum silage (Abdelhadi & Santini, 2006). We observed that the rumen MCP concentration increased when dairy cows ate sorghum silage diets. This indicates that the sorghum silage diet could make the rumen more effective in synthesizing MCP from NH_3_–N.

### 4.4 Amino acid profile of rumen fluid and functional bacterial groups

Our study detected an attractive phenomenon, which has not been reported in previous studies. The AA content in the rumen fluid of cows feeding sorghum silage is considerably increased (e.g., Thr, Ser, Gly, Val, Ile, Leu, Tyr, Phe, and Pro). The bacterial copy number results also showed that the abundance of protein‐degraded bacteria, [e.g., Bacteroides (Solden et al., 2017), *Prevotella* (Stevenson & Weimer, 2007), and *Prevotella ruminicola*] was increased in the rumen of cows fed a diet with sorghum silage. These may indicate that sorghum silage can boost the proliferation of rumen protein‐degrading bacteria. General bacteria and *Prevotella* are the most representative microorganisms in the rumen of ruminants (Wallace et al., 1997). General bacteria and *Prevotella* have protease activity that assimilates ammonia into MCP (Stevenson & Weimer, 2007). Our study found that feeding dairy cows a diet with sorghum silage can increase the abundance of *Prevotella* in the rumen, which is consistent with the lower NH_3_‐N and higher MCP content of cows from the sorghum silage group. A study has shown that Val, Leu, Ile, and Pro and hydrolyzed proteins containing these AAs can be converted into VFAs (acetic acid, isobutyric acid, dimethylbutyric acid, valeric acid, and isovaleric acid) and ammonia, which can stimulate the growth of rumen bacteria (Dehority et al., 1958). We found that cows in the sorghum silage group had higher ruminal fluid TAA and lower serum TAA content than that in the corn silage group. The correlation analysis of rumen fluid and serum AA profile shows that the content of most AAs in serum is negatively correlated with AA content in the rumen fluid. This is due to the abundance of protein‐degrading bacteria in the rumen of cows consuming the sweet sorghum silage diet increasing compared with the CON diet, which increased the degradation and consumption of AAs in the rumen. Furthermore, the decrease in AA content in the rumen fluid may be due to the AAs being transported to the blood and then provided to various tissues for use (Flythe & Andries, 2009).

### 4.5 Serum biochemical indicators and amino acid profile

The TP, GLU, TG, CHOL, and NH_3_L in the serum of all cows tested were within the normal range (Radostits et al., 2006). When animal health and protein metabolism are impaired, it is manifested as a decrease in serum TP content (Solaiman et al., 2010). Our research results show that the sorghum silage diet does not reduce serum TG content, which indicates that sorghum silage has no adverse effect on cow health or protein metabolism. Our results also show that feeding cow sorghum silage could reduce serum TG content. The high requirements of the mammary gland for lipids utilization may be met by an increase in free fatty acids, which is associated with a decrease in TG levels (Pysera & Opalka, 2000). Our study shows that the sorghum silage diet could increase the 4% FCM yield. Cows fed sweet sorghum silage have a higher demand for lipids in breast tissue due to higher FCM yield of 4%, resulting in a decrease in serum TG content. In our study, feeding a sorghum silage diet increased the serum NH_3_L content of cows. Serum NH_3_L is a crucial indicator of animal protein status and rumen performance (Hess et al., 2000). When the ammonia produced by rumen microbes decomposing protein exceeds the ammonia utilization by the microbes, excess ammonia can enter the blood. Our microbiological quantitative analysis results show that the sorghum silage diet can promote the proliferation of protein‐degrading bacteria, thereby increasing protein decomposition and resulting in an increase in blood NH_3_L content.

Our research indicated that cows fed sorghum silage had lower TAA content in the serum than those fed sorghum silage. In addition, the serum branched‐chain amino acids (BCAAs, Leu, Ile, and Val) were profoundly decreased in the cow fed a diet with sorghum silage. In this regard, Leu declined by 22.34% (CON vs. CS2) and 38.54% (CON vs. CS3), and Val decreased by 24.98% (CON vs. CS2) and 31.70% (CON vs. CS3). Previous research claims that serum BCAA content is a good indicator of AA absorption (Greter et al., 2008). Combined with our AA profile data of the rumen fluid, the content of BCAAs in the rumen fluid of dairy cows fed sorghum silage was higher than that of cows fed corn silage. The contents of Val, Leu, and Ile in the rumen are converted into isobutyric acid, isovaleric acid, and 2‐methyl butyric acid after oxidative deamination (Allison, 1978). In the present study, the absolute content of isobutyric and isovaleric acid in the rumen of cows consuming sorghum silage was higher than that of cows consuming corn silage. The correlation analysis of the AA profile of serum and rumen fluid also shows a significant negative correlation between the content of Leu and Val in serum and rumen fluid. This means that the increased content of BCAAs in the rumen fluid could produce more isobutyric and isovaleric acid. The lower content of BCAAs in the serum of cows fed sorghum silage diets might be associated with the microbial consumption in the rumen that uses BCAAs to synthesize isobutyric and isovaleric acid. An unusual event is that the BCAAs in the serum are significantly different, but we did not detect any difference in milk production and protein content among the treatments. Previous research claimed that BCAAs are essential for milk synthesis and account for more than 50% of the essential AAs in milk (Appuhamy et al., 2011; Mackle et al., 1999). Increased levels of Leu in the serum can stimulate rapamycin pathway targets in early lactation cows and induce increased protein synthesis, which has been reported in several species (Lynch & Adams, 2014; Torres‐Leal et al., 2011). However, the report claimed no milk protein depression when plasma Leu and BCAA concentrations decreased to 63 and 297 μM, respectively (Weekes et al., 2006). This study showed that although dairy cows ingested sweet sorghum silage diets caused a decrease in the concentration of BCAAs in plasma, the plasma Leu and BCAA concentrations of dairy cows in all treatments were higher than 63 μM and 297 μM, respectively. This indicates that although the sweet sorghum silage diet reduces the concentration of BCAAs in the plasma of dairy cows, the slight decrease in plasma BCAA concentration does not inhibit milk production and protein production.

## 5 CONCLUSION

Our results indicate that replacing corn silage with sorghum silage could increase milk yield and fat. Although the apparent nutrient digestibility was reduced by replacing corn silage with 75% sweet sorghum silage, the DMI and milk yield, the growth of rumen microbes, and the contents of rumen fluid AAs were increased. Since sorghum has lower water and soil fertility requirements and higher yields than corn, sorghum silage is feasible for dairy cows, and it is reasonable to replace it with 75% sorghum silage. In general, sorghum silage is expected to substitute corn silage in arid areas as the primary feed source for dairy cows.

## AUTHOR CONTRIBUTIONS

**xiaokang Lv:** Data curation (equal); formal analysis (equal); writing – original draft (equal). **Liang Chen:** Data curation (equal); formal analysis (equal). **Chuanshe Zhou:** Conceptualization (equal); funding acquisition (equal); writing – review and editing (equal). **Guijie Zhang:** Data curation (equal). **jingjing xie:** Data curation (equal); formal analysis (equal). **Jinhe Kang:** Writing – review and editing (equal). **Zhi Liang Tan:** Conceptualization (equal). **Shaoxun Tang:** Writing – review and editing (equal). **Zhiwei Kong:** Data curation (equal); formal analysis (equal). **Zixin Liu:** Data curation (equal); formal analysis (equal). **Zhiyan Du:** Data curation (equal).

## FUNDING INFORMATION

This work was supported by the Strategic Priority Research Program of the Chinese Academy of Sciences (XDA27040306); STS Project of the Chinese Academy of Sciences (CAS‐KFJ‐STS‐QYZD‐168); Major Special Project of Tibet Autonomous Region Science and Technology Plan (XZ202101ZD003N); and Science and Technology Department of Tibet (2020WK4002).

## CONFLICT OF INTEREST STATEMENT

The authors declare that no conflict of interest exists.

## ETHICS STATEMENT

The study received the approval of the Institutional Animal Care Committee, and all procedures involving animals were conducted following the guidelines on animal care of the Institute of Subtropical Agriculture, Chinese Academy of Sciences.

## Data Availability

Data may be provided following request to the corresponding author.
